# Causal inference: relating language to event representations and events in the world

**DOI:** 10.3389/fpsyg.2023.1172928

**Published:** 2023-09-18

**Authors:** Yipu Wei, Pia Knoeferle

**Affiliations:** ^1^School of Chinese as a Second Language, Peking University, Beijing, China; ^2^Department of German Studies and Linguistics, Humboldt-Universität zu Berlin, Berlin, Germany; ^3^Berlin School of Mind and Brain, Berlin, Germany; ^4^Einstein Center for Neurosciences Berlin, Berlin, Germany

**Keywords:** causal inference, event representation, implicit causality, expectation-driven processing, visual world paradigm

## Abstract

Events are not isolated but rather linked to one another in various dimensions. In language processing, various sources of information—including real-world knowledge, (representations of) current linguistic input and non-linguistic visual context—help establish causal connections between events. In this review, we discuss causal inference in relation to events and event knowledge as one aspect of world knowledge, and their representations in language comprehension. To evaluate the mechanism and time course of causal inference, we gather insights from studies on (1) implicit causality/consequentiality as a specific form of causal inference regarding the protagonists of cause/consequence events, and (2) the processing of causal relations. We highlight the importance of methodology in measuring causal inference, compare the results from different research methods, and emphasize the contribution of the visual-world paradigm to achieve a better understanding of causal inference. We recommend that further investigations of causal inference consider temporally sensitive measures and more detailed contexts.

## 1. Causal inference: the *sine qua non* of connecting events in language and the world in a meaningful way

Causal inference—speculating about causes and anticipating consequences (McKoon and Ratcliff, 1986; Hassin et al., 2002)—is a crucial step that enables people to interpret what has happened and predict what is coming next. This making sense of events is key to establishing coherent mental representations (Van Dijk and Kintsch, 1983; Zwaan, 1999). As argued by Radvansky and Zacks (2011), causal relations are central to event models. In language comprehension practice, for instance, causally connected events are better memorized and understood (Keenan et al., 1984; Trabasso and Van den Broek, 1985). The current paper reviews various sources of information that contribute to the basis of inference, and how and when causal inferences are made in language processing. We also suggest that temporally-sensitive methods would benefit the understanding of how causal inference unfolds during real-time language processing.

### 1.1. Events and event representations

*Events* are dynamic activities that occur in a fixed time and place (Zwaan, 1999, p. 95). They “convey a change in state in the world” (Kuperberg, 2021, p. 257). Events typically include components such as an agent, object, time, location, manner, and so forth (Altmann and Ekves, 2019). Language creates references to events in the world (Jackendoff, 2002, pp. 326, 342). For instance, a simple sentence such as *John jumped from the window* includes a verb *jumped* which denotes an action, a subject *John* as the agent of the event, and *from the window* as the event’s location. Such a sentence forms the linguistic representation of the event and its components (Altmann and Kamide, 2007). The mental simulations of these components, on the other hand, constitute the mental representation of the event (Altmann and Ekves, 2019). In this current review, we use the term *event representations* to refer to the latter sense of representations.

Generating and tracking event representations is important to understand sentences that describe events. As Zwaan (2004; *cf.* Barsalou, 1999) has argued, mental simulation of events is implicated in the process of language comprehension. Kukona et al. (2014) reported that their participants visually tracked the object location representation of an event (e.g., participants who were informed that sweetcorn would be put in a jar would gaze more at a jar than other objects when sweetcorn was mentioned), in line with the notion of mentally simulating or representing aspects of events. Similarly, in language processing, the manner and path of an event also appear to be represented in mental models along with the described action, as suggested by eye movements biased upward by the verb *jump* and downward by *crawl* (Kamide et al., 2016; see also Spivey and Geng, 2001; Lindsay et al., 2013).

The importance of events and establishing event representations is manifested in language production, comprehension, and learning. Production starts with the conceptualization of events and is followed by message planning and the linguistic formation of conceptual representations (Bock et al., 2004), which are also affected by event structures (Bunger et al., 2013). Specifically, the way speakers package event information (e.g., to mention the path of an event or not) is subject to the influence of event structure priming and language-specific experience in encoding events (Papafragou, 2011; Bunger et al., 2013). In language comprehension, events depicted in visual scenes help people rapidly establish thematic role relations and resolve thematic-role-relation ambiguities in real-time comprehension (Knoeferle et al., 2005; see details in Section 2). Events also matter for language learning. The theory of action-based language (Glenberg and Gallese, 2012) indicates that children can learn verbs more effectively when equipped with knowledge of the corresponding actions (p. 913). This claim is also supported by the finding that the acquisition age of certain actions (e.g., drinking) is strongly correlated with that of the corresponding verbs (Angrave and Glenberg, 2007, cited in Glenberg and Gallese, 2012).

### 1.2. Relations between events

Events are indexed in situation models representing the time and space in which they occur, the character(s) they involve, and their relation to the character’s goal and previous events (Zwaan et al., 1995, p. 292). One event can be connected to another via links that may be temporal, spatial, causal, and/or motivational (Zwaan, 1999, 2004). According to Zwaan (1999, 2004; see also Zwaan et al., 1995; Radvansky et al., 1998), such links contribute to different dimensions of an event-indexed situation model, which is composed of the temporal dimension (when the event happened), the spatial dimension (where the event happened), the causal-motivational dimension (why the event happened), and the protagonist dimension (who performed the event). Causal connections, as a core dimension of situation models, are built through causal inference (Zwaan and Radvansky, 1998, p. 172). Comprehenders perceive upcoming events as being caused or enabled by previous events in a situation model, and those events in a causal chain form the core of a coherent story (Bower and Morrow, 1990, p. 44; *cf.* Altmann and Ekves, 2019).

For example, when participants are interpreting an event as they perceive the world, they tend to believe there is a cause for the event (Noordman and Vonk, 1998, p. 193; see also Trabasso et al., 1984; Van den Broek, 1990a). In the sentence *John jumped from the window*, possible reasons can be inferred—the room was on fire, for instance. People also infer consequences from the event: John’s jumping from the window may generate the consequence that John hurt himself. Such causal inferences help people make sense of an event (see also McKoon and Ratcliff, 1986; Jackendoff, 2002). According to Radvansky and Zacks (2011, p. 613), entities and information connected in a causal chain are more likely to be stored and represented in one event model. As people comprehend language, their representations of narrated events such as *John jumped from the window*—including for example, the present object *window* as well as the action *jump*—are encoded in situation models and stored in long-term working memory (Ericsson and Kintsch, 1995). These stored and indexed events in individual integrated models are updated along with sentence comprehension in multiple dimensions (i.e., time, space, protagonist(s), causality, and intentionality; Zwaan et al., 1995; Zwaan, 2004; *cf.* Morrow et al., 1989).

At a discourse level, sentences and clauses that describe events tend to be connected in a sensible manner. Such *coherence in discourse* can be recognized through inferencing operations that establish relations between sentential units (Hobbs, 1979), such as cause–consequence, list, and problem–solution. These coherence relations are key to representing the world and its events (Zwaan et al., 1995) and organizing links between events (Radvansky and Zacks, 2017, p. 133). As such, they are crucial to constructing situation models in discourse representations (Trabasso et al., 1984; Myers et al., 1987; Bower and Morrow, 1990; Sanders and Spooren, 2007). As Sanders et al. (1993) have argued, understanding a discourse “means constructing a coherent representation” (p. 95; *cf.* Sanders et al., 2001; Oakley and Hougaard, 2008).

Among these discourse relations, causal relations are considered critical to sentence comprehension and representation (Keenan et al., 1984; Myers et al., 1987; Sanders and Noordman, 2000). For instance, Sanders and Noordman (2000, p. 46) reported that when a target sentence (e.g., *The construction of a subway in the center of Veendam will begin next year*) served as the solution of a problem described in the preceding context, such as *traffic accidents with pedestrians*, it was read faster than in the condition when it was just one of several listed constructions. Similarly, Myers et al. (1987) and Keenan et al. (1984) uncovered a linear relationship between reading speed and causal relatedness, finding that result sentences such as *The next day his body was covered with bruises* were read fastest after highly related sentences such as *Joey’s big brother punched him again and again*, medium after sentences with intermediate relatedness such as *Racing down the hill, Joey fell off his bike*, and were read most slowly after poorly related sentences like *Joey went to a neighbor’s house to play*.

In sum, causal inference helps to establish coherence between events and discourse segments (*cf.* Van den Broek, 1990b; McKoon and Ratcliff, 1992; Trabasso and Suh, 1993; Graesser et al., 1994), and such causal coherence facilitates comprehension. In the following we review what we view as part and parcel for accommodating causal inference in language processing with regards to the mental representations of events: We ask what information forms the basis of processing (Section 2), how and when causal inferences are made (Sections 3 and 4), and how they can be measured in specific paradigms (Section 5).

## 2. Sources of information in language processing

Comprehenders access multiple types of information in language processing, including verbal (connectives, verbs, nouns, prepositions, prosody, etc.) and non-verbal cues (e.g., visual context), both of which provide access to knowledge about the world and about events specifically (Tanenhaus et al., 1995; Altmann and Mirković, 2009; Mayberry et al., 2009; Kukona and Tabor, 2011). In reviewing the contributions of these different information sources—linguistic and non-linguistic—to language processing, Section 2 lays the foundation for the subsequent discussion on how and when causal inferences are computed in real-time processing (Sections 3 and 4).

Current linguistic input provides retrieval cues in short-term working memory and contributes to updating event information stored in long-term working memory (Zwaan, 2004). Verbs, for example, can be integrated rapidly into the representation of events and guide visual attention to entities in the real world. Previous work has shown that verbs not only trigger expectations^1^ for the objects that immediately follow them (Altmann and Kamide, 1999), but also predict a forthcoming object later in the sentence which fills the goal of a three-place verb (Kamide et al., 2003; see also argument activation of unaccusative verbs vs. agentive verbs in Koring et al., 2012). Kamide et al.’s (2003) study further revealed that verbs together with their grammatical subjects drive the anticipation of upcoming grammatical objects of the verb. For instance, the subject *the man* and the verb *ride* jointly enable expectations of the highly plausible object *motorbike*, whereas *the girl* and *ride* do not. In addition, verb tenses (e.g., *will drink* and *has drunk*) can activate the representation of objects depending on their temporal relevance (Altmann and Kamide, 2007), and structural cues (e.g., case-marking on pre-verbal nouns in a verb-final language like Japanese) also enable predictive processing (Kamide et al., 2003). These results suggest that the processes of inferring referents are mediated by various linguistic cues, which can help comprehenders anticipate possible arguments as the role fillers in event representations; such anticipation can be observed, for example, in the distribution of fixation proportions over objects that are time-locked to words in the utterance.

Connectives also shape expectations of upcoming events. Studies in discourse processing indicate that connectives may guide people’s attention to potential referents in visually-situated language processing. Mak et al. (2013) found that Russian connectives, which specify referent maintenance or shift, provide information for processing referents in discourse comprehension. For instance, the Russian connective *i* (*and*) is only used to connect two events that share the same referent; by contrast, *a* (*but*) often coordinates two separate topics that are associated with different referents. Native Russian speakers are sensitive to the difference between the two connectives and use them as cues to derive discourse expectations. When they encountered *a*, these speakers were more likely to shift attention to a different visual referent perceived as the agent of the event in the second clause. What is more, connectives that specify a more subjective causal relation, such as *kejian* (*therefore*) in Chinese and *dus* (*so*) in Dutch direct more attention to the speaker (as the person responsible for the subjective reasoning) compared to the more objective connectives *yin’er* and *daardoor*, which both mean *as a result* in their respective languages (Wei et al., 2019).

Another source of information in language processing is non-linguistic visual information, and its role in grounding language in relation to objects and events and their representations in memory. Tanenhaus et al. (1995) illustrated how immediate visual contexts influenced online sentence parsing using a “visual world” eye-tracking experiment in which participants listened to language while they inspected objects. In Tanenhaus et al.’s (1995) study, when hearing the temporally ambiguous auditory instruction *Put the apple on the towel in the box*, participants who saw only one apple in the visual context (plus an empty towel and other objects) were more likely to interpret *the towel* as the destination and thus anticipated its referent (the empty towel) as the destination of the action *put*. However, when participants saw two apples (one on a towel and the other on a napkin), they tended to interpret *on the towel* as a modifier of *the apple* and looked more at the apple on the towel than at the (empty) towel-destination when hearing *on the towel*.

The notion of *reference* is not restricted to entities but should also encompass events and actions, and one possibility is that not only isolated objects but also event depictions could be a useful source for real-time language processes like thematic role-assignment (Knoeferle et al., 2005; see also Jackendoff, 1983). When the thematic role of a sentence-initial subject was linguistically ambiguous shortly after participants had heard the verb (*Die Prinzessin wäscht…*“The princess (subject/object) washes…”), event depictions enabled participants to anticipate the role filler of the verb and its action referent—in this case, somebody depicted as being washed by a princess (Knoeferle et al., 2005).

Real-world properties of visual objects (e.g., their action-based affordances) also inform referential processing (Chambers et al., 2004). When hearing *Pour the egg in the bowl over the flour*, participants interpreted *in the bowl* as the modifier of *egg* when there were two liquid eggs in the visual context; but they tended to interpret *the bowl* as the destination of the action *pour* when there was one liquid and one solid egg. Evidence for this consisted of an immediate increase in visual attention to the empty bowl in the one-liquid-egg condition compared to the two-liquid-egg condition (Chambers et al., 2004; *cf.*, Chambers et al., 2002).

Real-world knowledge is activated during language processing and influences comprehension (McRae et al., 1998; Metusalem et al., 2012). For instance, stereotypical thematic role knowledge of the verb (e.g., the verb *spy on* takes *detective* as a stereotypical agent) is activated in real-time processing and influences anticipatory eye movements towards a role filler (Knoeferle and Crocker, 2006). Activated world knowledge also plays an important role in event integration and updating. As Zwaan (2004) proposed, “concordance with human experience,” which involves “continuity of time, space, and perspective,” influences how construal (a process that integrates activated “functional webs” in the “mental simulation of an event” at the clause level) is integrated into mental representations of discourse (pp. 40, 48). Graesser et al. (1994) stressed the importance of background knowledge stored in long-term memory for the construction of mental representations during comprehension, maintaining that mental representations of actions, states, and events, etc. can be “filled in inferentially by world knowledge” (p. 371), which is rooted in comprehenders’ social and perceptual experience (p. 372). Types of knowledge-based inferencing center on the causes of events, the referents of nouns/pronouns, characters’ emotions and beliefs, or the superordinate goals of characters (Graesser et al., 1994, p. 384).

In summary, comprehenders rely on linguistic input, non-linguistic visual information and real-world knowledge of events that can be derived from both language and the non-linguistic visual context. In the following sections, we examine how causal inference is established based on various sources of information (Section 3) and the timeline of when these cues come into play (Section 4).

## 3. The *what* and *how* of causal inference

According to Stewart et al. (2000), “The way in which people ascribe causes to events depends on the way in which a particular event is described” (p. 423). In this section, we review literature on referential processing in implicit causality/consequentiality, which highlights the role of verbs and connectives as important verbal cues (among others) for computing implicit causal inference (Section 3.1). We then extend to causal inference beyond the protagonist of cause/consequence relations in the second part of Section 3.1. Section 3.2 discusses the roles of connectives and world knowledge in establishing causal inference. In Section 3.3, we focus on how causal relations are established without explicit connectives.

### 3.1. Inferences concerning the protagonist and the causal relations in cause/consequence events

The well-known implicit causality effect can be seen as a special type of causal inference that surfaces in the resolution of a pronoun referent serving as the protagonist/agent of the cause of a previous event. For example, a sentence such as *Sally frightened Mary* denotes an event that involves two characters. When this sentence continues with a connective *because* followed by the ambiguous pronoun *she* in the second clause, participants need to figure out which character, Sally or Mary, is the referent of *she*. This character will then be the protagonist in the next event which explains why Sally frightened Mary (Sally is preferred in this case given the verb bias). Given the information encoded by the verb, comprehenders may develop expectations of the protagonist of the event and also infer its cause in the process (Au, 1986). This *implicit causality bias* is described as language users’ tendency to “attribute the causes of events described by some interpersonal verbs either to the subject or to the object of the clause containing the verb” (Cozijn et al., 2011a, p. 382; see also: Garvey and Caramazza, 1974; Caramazza et al., 1977; Garnham et al., 1996).

Implicit causality verbs differ in terms of the biases they trigger. Verbs such as *frighten*, *surprise*, or *anger* (see example 1a) bias comprehenders to see the events in the next clause as performed by the first character (Sally) whereas verbs such as *like*, *hate*, or *admire* (example 1b) induce a preference for Mary, the second character (Garvey and Caramazza, 1974; Koornneef and Van Berkum, 2006; Cozijn et al., 2011a). Alternatively, some have categorized implicit causality verbs as either subject-or object-biased based on the syntactic position of the character that the verb appears to favor (Hartshorne and Snedeker, 2013; Hartshorne, 2014).

(1) a. Sally frightened Mary because she…

b. Sally feared Mary because she…

(Hartshorne and Snedeker, 2013, p. 1474).

Alongside implicit causality, *implicit consequentiality* has been introduced and examined by Stewart et al. (1998) and Commandeur (2010). The first of these studies found that people engaged in a production task exhibited consequentiality biases when continuing sentences with the form *Because NP1 Verb-ed NP2*… For instance, the verb *annoyed* biased people to continue the sentence with a focus on the NP2, who had suffered from the consequence of being annoyed. Such implicit consequentiality was confirmed by empirical evidence showing that reading times increased when situations were inconsistent with expectations driven by implicit consequentiality (Stewart et al., 1998).

Crinean and Garnham (2006) treat both implicit causality and implicit consequentiality as matters of expectation in terms of whether a cause or consequence is generated and which character is involved. They argue it is the thematic role of event participants assigned by a verb that defines implicit causality and consequentiality associated with the verb (Crinean and Garnham, 2006). The stimulus-experiencer verbs, such as *annoy* and *amaze*, take the stimulus (NP1) as the cause and the experiencer (NP2) as the consequence (Crinean and Garnham, 2006; *cf.* Au, 1986). Sentences with main clauses such as *John amazed Bill because*… would explicitly (*via because*) generate an expectation for a cause, and *John* (NP1, the stimulus and the cause) should be the focus of the subordinate clause. In contrast, if the sentence starts with *John amazed Bill so*…, the subordinate clause is explicitly specified as the consequence of *John amazed Bill*. Therefore, *Bill* (NP2, the experiencer and the consequence) should be the focus of the subordinate clause.

The protagonist of the cause/consequence such as *John* and *Bill* takes a salient position in the “who-did-what-to-whom” event structure (Papafragou, 2011), and this prominence means it is retrieved more rapidly than other event components. The bias towards a certain protagonist of the upcoming event can be seen as an indication of how a previous event is stored in, and retrieved from memory and how it is connected to an upcoming event.

However, the tendency to anticipate causes or consequences does not just involve a particular entity or a character as implicit causality/consequentiality studies have demonstrated. An event or a situation (which can but does not have to represent a cause or consequence) also includes locations (Jackendoff, 2002; Kukona et al., 2014), sources and goals (Dowty, 1989), actions (Knoeferle et al., 2005), as well as speakers (Knoeferle and Kreysa, 2012; Carminati and Knoeferle, 2013). In fact, studies have shown that implicit causality effects can be modulated by the temporal structure of events in prior discourse (e.g., the temporal distance between cause and consequence events; Dery and Bittner, 2016; *cf.* Bott and Solstad, 2014), demonstrating a close relationship between referent resolution biased by implicit causality and event comprehension. As put by Pickering and Majid (2007), implicit causality “provides an abstraction of the type of reason that is most likely to be provided for the event, and indicates which entity the reason tends to be about” (p. 786). For example, in the sentence *David apologized to Linda…*, apart from expectations about the likely protagonist of the cause event (David in this case), comprehenders may also anticipate the reason for the event, like David apologizing (for instance, that what David did requires an apology).

To test how implicit causality verbs affect people’s expectations of the potential cause or consequence of an event (causal inference in a broader sense, not just inference of the protagonist), Rohde and Horton (2014) first trained participants to associate spatial locations on a Y-shaped “tube” with two ends—the left for *cause* and the right for *consequence*. The *cause* and *consequence* continuation of the prompts were combined with the cause/consequence biasing verb conditions as illustrated in examples (2) and (3). Participants heard prompt sentences with either implicit causality verbs (e.g., *admire, please, scold*) as in example (2) or transfer-of-possession verbs (e.g., *hand, give, ship*) as in example (3). Implicit causality verbs were assumed to bias the continuation to a cause explaining the eventuality in the prompt; transfer-of-possession verbs were expected to bias the continuation to a consequence^2^ caused by the prompt. Participants’ gaze directions were measured as they listened to the sentences.

(2) Implicit causality prompt (cause bias): Arthur scolded Patricia in the hallway.

a. Cause continuation: She had put thumbtacks on the teacher’s chair.

b. Consequence continuation: He then sent her to the principal’s office.

(3) Transfer-of-possession prompt (consequence bias): Heidi shipped Eric a package.

a. Cause continuation: She thought he’d like some cookies from home.

b. Consequence continuation: He wrote her a thank you note.

(Rohde and Horton, 2014, p. 677).

Rohde and Horton (2014) found that the *cause* location (represented by the left end of the Y-shaped tube) was preferred over the *consequence* location (the right end of the tube) when hearing the implicit causality verbs; this preference was reversed under the transfer-of-possession verbs condition. The researchers drew two conclusions from their findings: First, when cued by linguistic input, comprehenders may infer cause and consequence as forthcoming events, not just the protagonist; second, the verb type determines whether a cause or a consequence is inferred.

Studies on implicit causality have demonstrated that the information encoded in the semantics of the verbs imposes constraints on causal attributions (Crinean and Garnham, 2006; Bott and Solstad, 2014; Van den Hoven and Ferstl, 2018). Such attribution introduced by the verb can be viewed as enriched *via* the meaning relations that connectives establish (Koornneef and Sanders, 2013; Xu et al., 2019; Lyu and Wang, 2022). For instance, implicit causality effects introduced by verbs may only hold for sentences with the connective *because*, but not for those with *but* or *and* (Koornneef and Sanders, 2013). Apart from connectives, other linguistic factors including order of mention (Järvikivi et al., 2005, 2017), semantic properties of nouns (Corrigan, 2001; Frenzel et al., 2015), ontological constraints (Bott and Solstad, 2021), as well as discourse structure (Bott and Solstad, 2014; Dery and Bittner, 2016) also shape such inference.

### 3.2. Causal inference elicited by connectives and world knowledge

Connectives link events and function as retrieval cues that foreground causal relations between events (Zwaan and Radvansky, 1998). In real-time processing, causal connectives facilitate readers’ online processing of sentences that express relations between events (Millis and Just, 1994; Van Silfhout et al., 2014, 2015; Canestrelli et al., 2016). For instance, readers benefit from the presence of connectives (e.g., *moreover*, *after*, *because*) when comprehending narratives, showing faster reading times of subsequent content and shorter rereading times of prior text compared to conditions without connectives (Van Silfhout et al., 2015).

The role of connectives interacts with that of world knowledge in causal inference. For example, the link between *it rains* and *the street is wet* could be introduced by a causal connective *so* or be derived from world knowledge (a bridging inference). As argued by Kuperberg et al. (2011), to establish causal coherence between two events, it is necessary to infer the meaning of implicit information using real-world knowledge. When the world knowledge of events contradicts the coherence relations asserted by linguistic cues, Xu et al. (2015) found increased processing efforts in sentence interpretation. In their event-related brain potential (ERP) study, Xu et al. examined sentences containing either a causal or concessive connective, both of which have a causal assumption. When the causal assumption is not satisfied by real-world knowledge, they found an N400^3^ effect followed by a P600 effect (late positivity) for the *because*-incongruent sentences and an N400 effect followed by a late negativity effect for the *although*-incongruent sentences compared to the congruous condition; this suggests increased processing difficulties for both connective conditions but different neural mechanisms of re-establishing coherence relations expressed by different connectives (*cf.* Lyu et al., 2020 and Xu et al., 2018 for comparison between concessive and causal relations using eye-tracking, and Xu et al., 2022 for fMRI evidence).

Expectations derived from real-world knowledge can be changed by the choice of connectives. In an ERP study, Xiang and Kuperberg (2015) investigated the role of the concessive connective *even so* in shifting expectations inferred from world knowledge. For instance, for the two sentences *Elizabeth took the test and aced it* and *She went home and celebrated*, the second sentence meets the expectation of a world knowledge model activated by the first sentence (Xiang and Kuperberg, 2015, p. 649). However, this expectation is reversed by inserting *even so* between the two sentences, which, according to Xiang and Kuperberg (2015), constructs a discourse model that opposes real-world knowledge. The incoherence between the situation depicted in the second sentence (which is consistent with the real-world knowledge) and the discourse model created by *even so* (inconsistent with the real-world knowledge) led to larger N400 effects compared to conditions coherent with the discourse model constructed by *even so* but against the world knowledge model (*Elizabeth took the test and failed it. Even so, she went home and celebrated*; Xiang and Kuperberg, 2015, p. 649). It is concluded from this study that linguistic cues may alter the causal expectations created by world knowledge.

This interpretation is consistent with ERP studies on the interplay between the semantic meaning of *after/before* and the real-world knowledge about the temporal order of two events, which is closely linked with causal inference (Münte et al., 1998; Nieuwland, 2015). When the temporal structure encoded by *after/before* is inconsistent with the real-world knowledge of event ordering, larger N400s (Nieuwland, 2015) and greater left-anterior negativity (LAN, i.e., increased mean-amplitude negativity in the left frontal regions which is associated with more demanding processing; Münte et al., 1998) were elicited.

### 3.3. Causal inference in the absence of explicit connectives

Without explicit causal connectives, causal inference can also be elicited by world knowledge that is activated during sentence processing. Singer et al. (1992; see also Singer, 1993) found that causal bridges between sentences such as *Sharon took the aspirins. Her headache went away* can be validated with reference to world knowledge (e.g., *Aspirin relieves pain*; p. 507). They tested this hypothesis with a series of reading experiments and found that the response time required to answer questions about world knowledge (e.g., *Do aspirins relieve headaches*?) was indeed shorter after reading sentences linked causally compared to temporally linked sentences. This suggests that processing may speed up following the pre-activation of world knowledge associated with causal relations. World knowledge of an event’s typicality also influences causal reasoning (Corrigan, 1992; Simner and Pickering, 2005; see discussion on the timeline of accessing event knowledge in Section 4.3). More typical events were more likely to elicit multiple causal attributions, i.e., to attribute causality to an event participant (Corrigan, 1992, p. 364). Moreover, when the agent in the context was typical, people produced more continuations of consequences than when the agent was non-typical (Simner and Pickering, 2005).

Cozijn et al. (2011b) differentiated two separate processes of causal inference: *propositional integration*, by which relations are established between clauses or sentences, and *world-knowledge inference*, which refers to a process of “deriving the general causal relation and checking it against the reader’s world knowledge” (p. 498; *cf.* text-based and knowledge-based connections, Coté et al., 1998). The integration process occurs at the start of the second clause and is facilitated by the presence of the connective *because*, which reduces reading times. However, the inference process occurs later in the subordinate clause when sufficient information for world-knowledge inference is provided, increasing reading times at the end of the sentence (Cozijn et al., 2011b; for detailed discussion of when world-knowledge is accessed, see Section 4.3).

Visual cues also modulate inferential processing. Cohn and Kutas (2015) suggested that in processing visual event sequences, a surprised face presented visually implied an off-panel event, introducing more information that triggers inferences—as manifested by larger positivity effects in ERPs compared to a condition without such a visual cue. Not much research has been done, however, to explicitly examine the role of visual cues in language-based causal inference. A pioneering study by Van Veen (2011) evaluated young children’s understanding of subjective and objective causal relations *via* a *preferential looking eye-tracking* study, in which possible causes were depicted (rather than implied by language). Participants viewed two scenes in one display while listening to a sentence expressing either an objective consequence-cause relation or a subjective claim-argument relation. In the former condition, participants heard a sentence describing a consequence event (e.g., *The cup lands on the ground*), and the target picture (depicting the cause of the event) showed a man causing the cup to fall. In the latter (subjective claim–argument) condition, the verbal sentence was an evaluative claim (e.g., *The man is tired*), and the target picture (depicting the argument for the claim) showed a man lying in bed. Both the two- and three-year-olds cast adult-like anticipatory glances toward the target picture depicting the cause of an objective consequence–cause relation. However, for the subjective claim-argument relation, only the adults and the three-year-old (but not the two-year-old) children looked significantly more at the target picture than at the alternative image (which showed the same character in an event irrelevant to the claim), thereby indicating their ability to perform subjective reasoning.

To sum up, Section 3 has reviewed the literature concerning what sources of information contribute to causal inference. We linked reference resolution guided by implicit causality/consequentiality to causal inference of cause/consequence as a complete event and evaluated the role of various sources of information in guiding causal inferencing.

## 4. The *when* of causal inference

The debates about the time course of causal inference concern at least three aspects, each of which constitutes a piece of the puzzle to when causal inference is computed, namely, when causes/consequences are generated (Section 4.1), at what point in time linguistic cues that establish coherence relations are processed (Section 4.2), and when people make use of events and event knowledge (i.e., world knowledge of events) (Section 4.3). Recognizing that the three aspects may not be independent of each other (Hald et al., 2007; Otten and Van Berkum, 2007; Van Dijk, 2019), we reviewed each to gain better insight into the time course of causal inference.

### 4.1. When causes/consequences are inferred

Early debates on the processing of causal relations centered on whether causes and consequences are generated online in reading comprehension. Some of this research specified limited circumstances for the online generation of causal inference, such as in highly constraining contexts or when readers aim to answer a question related to the text (Noordman et al., 1992). Converging evidence from different paradigms (sentence reading time, lexical decision, etc.) indicates that causes can be inferred online (Keenan et al., 1984; McKoon and Ratcliff, 1986; Myers et al., 1987; Van den Broek, 1990b; Singer et al., 1992). For instance, when a preceding sentence specifies a cause closely related to the following sentence, the second sentence takes shorter reading times than when the prior context is less causally related, indicating that relations between text segments are established online during reading (Keenan et al., 1984). Using a lexical decision task (similar to McKoon and Ratcliff, 1986), Magliano et al. (1993) measured the difference in lexical decision latency for an inference word vs. an unrelated word after participants had read passages whose causes needed to be inferred. Faster lexical decision times for inference words encoding the causes suggested that these causes could be inferred online when sufficient time to read the narratives was available.

Hassin et al. (2002) proposed and tested the *Spontaneous causal inference* hypothesis, which suggests that the cause of an event (a scenario) is spontaneously inferred. Hassin et al.’s study relies on the logic that as an event is represented in memory, the cause (e.g., *being pickpocketed*) of the event (*her wallet was missing*) is spontaneously inferred and encoded in memory along with the narrated event. If this holds, the causal cue *pickpocket* would facilitate event recall. In line with this, the sentence *After spending a day exploring beautiful sights in the crowded streets of New York, Jane discovered that her wallet was missing* was better recalled when a causal cue such as *pickpocket* was given than when a word repeated from the original sentence such as *sights* was provided.

The results on consequence inferencing are more mixed. McKoon and Ratcliff (1986) examined how participants inferred consequences in an immediate word recognition test. Participants first read a so-called “predicting” sentence such as *The director and the cameraman were ready to shoot closeups when suddenly the actress fell from the 14th story* (p. 83) and they were then prompted to recall whether or not a test word (e.g., *dead*) was actually mentioned in the predicting sentence. Their response times were then measured. Participants were slower to reject the test word when it represented a possible consequence of the predicting sentence than when it did not (e.g., *dead* after a figurative use of *fell upon* as in *Suddenly the director fell upon the cameraman, demanding close-ups of the actress on the 14th story*, p. 83). These results were interpreted as evidence of an online inference process: “…the predicted event was inferred during reading of the sentence and stored in the memory representation of the sentence” (McKoon and Ratcliff, 1986, p. 84), thereby making it harder to refute the test word representing the predicted consequence.

However, using a similar lexical decision measure, Magliano et al. (1993) found no evidence for the online generation of consequences. Singer and Ferreira (1983) measured response times to questions about consequences such as *Did the spy burn the report?* after participants had read a story containing inference antecedents (e.g., *The spy threw the report into the fire*). The long response time to the forward inference questions indicated that consequences were not inferred during story reading but rather delayed. Notably, research methods seem to influence whether inferences of consequences are found online. For instance, in Potts et al.’s (1988) study, the lexical decision task suggested that subjects inferred possible consequences while they read, as evidenced by their faster reactions to the inference word after reading a text predicting the consequence vs. a control text which did not activate an inference. However, when the same authors used a task that contained the same materials and conditions but required subjects to name the inference word, the ability to infer consequences was not observed (i.e., there was no difference in the word naming time between the predicting and the control conditions).

### 4.2. When linguistic cues are processed

With the development of finer-grained comprehension measures, researchers are able to explore the precise time course of linguistic processing involved in causal inference. For instance, they could determine whether causal relations are established integratively at the end of a sentence with all information available or incrementally in a word-by-word fashion. An *integration account* was proposed and tested by Millis and Just (1994) to evaluate the processing of discourse relations. The model suggests that readers reactivate and integrate information from the first clause only at the end of the second clause, especially when a connective is present (Millis and Just, 1994). This reactivation and integration process is indicated by longer reading times in the final part of the second clause. Similar arguments that the integration of clauses happens at the end of sentences were previously made by Kintsch (1988) and Kintsch and Van Dijk (1978).

However, plenty of evidence opposes the integration account in the field of discourse processing. For instance, Traxler et al. (1997) used an eye-tracking reading study to test the processing difference between objective causal relations (cause-consequence relations, e.g., *Heidi felt very proud and happy because she won first prize at the art show*) and subjective causal relations (claim-argument relations, or diagnostic relations in their term, such as *Heidi could imagine and create things because she won first prize at the art show*). The latter type of relation is hypothesized to be harder to process as it requires complex inference of subjective beliefs. If discourse relations are processed integratively, the processing difficulty associated with subjective causal relations should appear towards the end of a sentence, where the meanings of two clauses are integrated. However, if readers process clauses incrementally, such processing difficulty (represented by a reading delay) should be observed as soon as readers must adopt subjective reasoning, i.e., before the end of the sentence. In fact, the processing delay was observed well before the final region: In the example above, readers slowed down around *first prize* or even earlier (Traxler et al., 1997), providing clear evidence for the incremental account. Canestrelli et al. (2013) also compared subjective and objective relations, but in a language (Dutch) that has different connectives specific to each type of causal relation: *want* and *omdat* (both meaning *because*) to express subjective and objective causal relations, respectively. Readers were sensitive to the difference between the two types of causal relations, just as Traxler et al. had found: Longer reading times were associated with the subjective than objective relations. Most importantly, the difference in reading times occurred right after the specific subjective connective, thereby corroborating the incremental account of discourse processing.

In recent decades, predictive or expectation-driven language processing has received much attention (Pickering and Garrod, 2013; Pickering and Gambi, 2018). Readers receive language input and predict the phonological forms of incoming words (DeLong et al., 2005), the forthcoming grammatical objects (Altmann and Kamide, 1999; Kamide et al., 2003), or the syntactic structure of sentences (Levy, 2008). A large amount of research has substantiated the expectation-driven or predictive nature of language processing from various theoretical perspectives, ranging from probabilistic information-theoretic accounts (Hale, 2006; Levy, 2008), to simple recurrent networks (Altmann and Mirković, 2009; originally proposed by Elman, 1990), to forward modeling (Pickering and Garrod, 2013; first raised by Wolpert, 1997). In line with these expectation-driven processing models, it has been argued that human brains are predictive and driven by top-down expectations (Clark, 2013; Heilbron et al., 2022).

The expectation-driven account of language processing is not restricted to predicting upcoming words and sentence content. Language users also establish discourse relations between sentential units in an expectation-driven fashion, the so-called coherence-driven expectations in discourse processing (Trabasso and Suh, 1993; Rohde et al., 2011; Rohde and Horton, 2014; Xiang and Kuperberg, 2015; Scholman et al., 2017). Scholman et al. (2017) showed that comprehenders are sensitive to the structure of discourse representations signaled by discourse markers such as *on the one hand*, which they use to establish the expectation of contrast. In real-time processing, this expectation can be established immediately and maintained across multiple sentences. The extent to which the content inserted between *on the one hand* and the paired cue phrase *on the other hand* satisfied readers’ expectations impacted the time needed to process the latter phrase (Scholman et al., 2017).

The generalizability and reliability of the expectation-driven account of language processing have been challenged (see the review by Huettig and Mani, 2016), especially for bilinguals using their second language (in reading comprehension: Martin et al., 2013; in auditory comprehension: Ito et al., 2018) and with low-literacy subjects (Mishra et al., 2012). Additionally, verb-driven anticipatory looks to the target object—used in many studies to argue for expectation-driven processing—appeared absent when realistic and dynamic scenes were presented to participants (Andersson et al., 2011; De Almeida et al., 2019; but see counterevidence from Heyselaar et al., 2020). Nevertheless, for young proficient monolingual language users at universities, there seems to be good evidence in favor of at least partially expectation-driven language processing, which in turn can be viewed as a stepping stone to understanding the timeline of causal inference.

### 4.3. Making use of events and event knowledge

Events and event structures (“who-did-what-to-whom”), as part of world knowledge, play a key role in expectation-driven language processing (e.g., McRae and Matsuki, 2009; Venhuizen et al., 2019; Kuperberg, 2021). For instance, events and event structures can be used by comprehenders to pre-activate linguistic elements (syntactic, semantic, phonological, and orthographic information, Kuperberg and Jaeger, 2016). Event knowledge activated by linguistic cues (e.g., verbs, nouns, verb tense, etc.) modulates expectations of syntactic structures (Trueswell et al., 1993; MacDonald, 1994; Garnsey et al., 1997), the anticipation of upcoming concepts (McRae and Matsuki, 2009), and the priming of event locations (Ferretti et al., 2007). In this section, we review findings on when event information and event knowledge are accessed by language users when they compute causal inference.

When comprehending frequently-experienced event sequences, a cause is anticipated once the effect is provided and vice versa, highlighting the key role of predictability in integrating the sequence of causal events (Zwaan, 2004, p. 50). Duffy (1986) examined three expectation models that approach sentence processing at an event level: models of *focus* (first proposed by Garrod and Sanford, 1977), *prediction* (Olson et al., 1981) and *activation* (Haberlandt and Bingham, 1978). All three of these contrast with the *backward-inference-only* model which allocates a diminished role to expectation. In the three expectation models, it is hypothesized that reading is facilitated when a subsequent sentence fulfills expectations from prior contexts. However, each model differs from the other in its definition of *expectation*. The activation model defines expectation as “related concepts activated in semantic memory,” which is generated unselectively for every sentence (p. 210). The focus model defines expectation as a focus of attention on particular expectation-generating sentences that need to be followed up (p. 209). The prediction model also considers expectation as generated selectively for certain sentences, but unlike the focus model, it assumes that the generated expectation contains a specific proposition that represents a predicted event. When Duffy (1986) tested each of the three, only the focus model received consistent support. That is to say, recipients’ expectations are more likely to focus on sentences that require causes or consequences in later sentences, but are not generated after every prior sentence; and such expectations do not contain specific forms of anticipated events.

Gaining a better understanding of when in time event knowledge is activated is also important for arriving at a better understanding of the time course of causal inference, since inferences are likely verified against event knowledge from prior discourse (Singer et al., 1992). Such activation of real-world event knowledge is sometimes required to establish more complex inferences when linguistic input is otherwise insufficient (Long et al., 1990). One key question is whether real-world knowledge is activated only after incoherence between events has been perceived, or whether it is already enabled at the earliest stage long before any gaps in coherence are detected.

Some studies show that the effect of plausibility information (how likely the sentence is) based on event knowledge is *delayed* in thematic role assignment (Rayner et al., 2004; Warren and McConnell, 2007), recovery from the misanalysis of garden-path sentences (Traxler et al., 1998) and the resolution of discourse roles (Garrod and Terras, 2000). However, other researchers—such as Matsuki et al. (2011) who used more typical items to test thematic role assignment than the studies mentioned above in that context—argue that the effect of such plausibility information is immediate or even appears in parafoveal reading (Murray, 1998; Murray and Rowan, 1998).

In an ERP study, Kuperberg et al. (2011) examined how quickly event knowledge became available in causal inference. They compared three scenarios that differed in the degree of causality linking a target sentence (e.g., *She had sunburn on Monday*) with its prior context: a context that was highly related in terms of causality (*Jill had very fair skin. She forgot to put sunscreen on*), an intermediately related context (*Jill had very fair skin. She usually remembered to wear sunscreen*), or a causally unrelated context (*Jillʼs skin always tanned well. She always put on sunscreen*). The amplitude of N400s in response to the critical word *sunburn* in the target sentence reflected the effect of event knowledge activation in causal inference. The researchers hypothesized that without the prior activation of event knowledge (i.e., when no causal inference occurred before the critical word), the N400 amplitude in the intermediately related and unrelated conditions would be the same, indicating that a coherence gap was only detected at the critical word in the last sentence. If event knowledge was activated beforehand, however, there would be a smaller N400 amplitude in the intermediately related condition, similar to that of the highly causally related condition. The results leaned towards the latter account: The activation of event knowledge in causal inference is immediate.

## 5. Measuring causal inference

Findings that support views of language processing as incremental or predictive (at least partially) derive from online time-sensitive methods, highlighting the importance of methodology in examining causal inference. As Traxler et al. (1997) have suggested, methodological differences might account for disparate results and resulting distinct accounts of language processing. In this section, we review various empirical paradigms applied to measure causal inference including retrieval paradigms, brain activity measures such as ERPs and fMRI, and eye-movement measures of reading times and visual world processing. The time course of processing implicit causality is taken as an example to show how evidence from different paradigms (retrieval paradigms, ERP measure, reading and visual world paradigm eye-tracking) vary and accordingly prompt researchers to formulate distinct accounts. We confine ourselves to discussing details of insightful tasks such as self-paced reading (see Stewart et al., 2000), sentence completion paradigms (Simner and Pickering, 2005; Majid et al., 2007) and dual-task paradigms (Morera et al., 2017) in a single review.

### 5.1. Retrieval paradigms

Retrieval paradigms (including the cued-recall test, immediate recognition test, and word recognition test) have been widely applied to examine causal inference in comprehension. A cue word that indicates the cause of a sentence can facilitate sentence recall (Hassin et al., 2002; see details in Section 4.1). One explanation of these findings is that performances in cued-recall tests reflect how information is encoded in memory. When a sentence representing an event is encoded in memory, the traits/actions/physical events that serve as the causes or the consequences of the event in the sentence may also be represented. This means that a cue word (cause or consequence) might activate the whole cause-consequence scenario and that is why the retrieval of the given sentence is facilitated. However, cued-recall tests cannot reveal whether causes are inferred at the encoding stage or at retrieval.

Another type of retrieval paradigm, the word recognition test (or lexical decision test, McKoon and Ratcliff, 1986), provides a closer look into memory representations. The test assumes that if a consequence of an event is encoded in memory alongside the event at the encoding stage, a probe word representing the consequence would be difficult to falsify even if it had not been explicitly mentioned. With manipulations of the time interval between the sentence and the presentation of probe words, such a paradigm has been used to measure both immediate and long-term memory retrieval. Additionally, probe words can be inserted at different points in the sentence in recognition tests to give a semi-online measure of sentence comprehension at different processing stages (McDonald and MacWhinney, 1995; Garnham et al., 1996).

For example, McDonald and MacWhinney (1995) conducted to the best of our knowledge the first study to examine the timeline of the effects of implicit causality using retrieval paradigms. Their findings did not rule out the integration (Millis and Just, 1994) or the incremental accounts (Traxler et al., 1997). In the experiment, as people heard spoken sentences (e.g., *Gary* “*amazed*” (subject-bias verb) or “*admired*” (object-bias verb) *Alan time after time, because he was so talented*), they were probed with words on a screen (either the first name *Gary* or the second name *Alan*) at four sentence positions, and were asked to decide whether the word had been mentioned in the sentence. The participants reacted to the first-mentioned name faster in general. However, the *type* of implicit causality verb interacted with the first-mention bias at two positions: immediately after the pronoun and at the end of the second clause (McDonald and MacWhinney, 1995). Thus, one might argue that the influence of implicit causality information on anaphor resolution surfaced at these two positions.

Using a similar probe recognition paradigm, Garnham et al. (1996) tested participants when they were reading NP1-verb sentences such as *Walter apologized to Ronald this morning because he*… and NP2-verb sentences like *Jeff believed Paul yesterday because he*… with continuations about a protagonist either congruent or incongruent with the expected one. A proper name (either NP1 or NP2) was probed after the pronoun and at the end of the sentence. Garnham et al. (1996) found a stronger and more reliable implicit causality effect (faster and more accurate reactions to the probed name under the congruent than incongruent conditions) at the end of the sentence (i.e., the integration stage), compared to the point after the pronoun (Garnham et al., 1996; see also Stewart et al., 2000). This finding supported the *integration* rather than the *incremental account*, which would predict faster and more accurate reactions to the probed name immediately after the pronoun.

### 5.2. Measures of brain activity

Language processing is closely linked with brain activities, which can be observed with electroencephalographic (EEG) and functional magnetic resonance imaging (fMRI) measures (Kutas and Hillyard, 1984; DeLong et al., 2005; Willems et al., 2016). EEG provides a millisecond-resolution measure of neural activities, which is considered more direct and instantaneous in measuring language processing (Luck, 2013). FMRI signals, in contrast, are reflections of delayed and secondary blood flow responses coupling neural activities on the time scale of seconds (Heeger and Ress, 2002). However, fMRI reveals a higher spatial resolution of the brain areas activated during processing in comparison to the ERP measure.

#### 5.2.1. Event-related brain potentials (ERPs)

Brain waves recorded by electroencephalogram (EEG) in language research are analyzed in a time-locked fashion to the presentation of a stimulus, hence the name event-related brain potentials (ERPs). As discussed in Sections 3.2 and 4.3, ERP effects such as N400 and P600 serve as indexes that reflect how various sources of information are put together as participants form the mental representations of a situation or relations between events during language processing (Kuperberg et al., 2011; Xiang and Kuperberg, 2015; Xu et al., 2015).

ERP evidence shows that causal inference affects lexical processing in subsequent sentences (manifested by N400s) and requires extra efforts associated with updating mental models (P600s) (Burkhardt, 2006). St. George et al. (1997), for instance, found attenuated N400 effects (indicating reduced efforts in semantic integration) for words in the subsequent sentence preceded by a context that triggered inferences (see also in Davenport, 2014; *cf.*, Yang et al., 2007); Kuperberg et al. (2011) claimed that such N400 attenuation was modulated by the strength of causal inference required to connect the final sentence with the previous discourse. Late positivity effects (P600s) were found to be related to integrating new information to the mental representations established by the prior context (Sitnikova et al., 2008; Hoeks and Brouwer, 2014). In inferential processing, particularly, enhanced P600s paired with conditions that demand drawing inferences to reach coherence, such as between *a student was killed* in the context and *pistol* in the subsequent sentence, compared to the more obvious relations between *being shot* and *pistol* (Burkhardt, 2007; *cf.* Cohn and Kutas, 2015 for visual event processing).

An example of how ERP studies reveal a different view on the processing of causality in comparison to earlier retrieval paradigms comes from Van Berkum et al. (2007). In their study, implicit causal inference in reading was measured. Embedded in a second clause was an ambiguous pronoun (*he*), which was either consistent with the gender of the expected protagonist of the second clause given the implicit causality information in the first clause (e.g., *David apologized to Linda because he…*) or inconsistent with it (e.g., *Linda apologized to David because he…*). They found that a rapid differential ERP effect emerged around 400-700 ms after the onset of the pronoun when pronoun gender was inconsistent (vs. consistent) with the implicit causality verb bias. In the ranges of 400–500 and 600–700 ms, there was a significantly smaller mean amplitude under the inconsistent condition than that in the bias-consistent condition, which was interpreted as a referentially induced P600 effect. The ERP effects suggest that readers immediately verify whether the newly encountered pronoun fits the situation model they already formed, lending support to the incremental account (Van Berkum et al., 2007).

#### 5.2.2. Functional magnetic resonance imaging (fMRI)

Functional magnetic resonance imaging (fMRI)^4^ studies complement ERP-evidence with information on the brain regions involved in language processing and the extent to which specific regions in the brain are activated. Different brain regions, as established in the literature, can be implicated in cognitive processes such as semantic retrieval (Hagoort et al., 2004), inhibitory control (Vitale et al., 2022), theory-of-mind reasoning (Amodio and Frith, 2006; Carrington and Bailey, 2009) among others. Evidence from fMRI contributes to the current topic by revealing the brain networks underlying different types of inferences (see Feng et al., 2021 for a meta-analysis review of fMRI studies on various types of inferences). Particularly, Ferstl and Von Cramon (2001, 2002) located the main active area for establishing causal coherence, the left frontomedian cortex, which is responsible for elaborative inferential processing. Kuperberg et al. (2006) examined three scenarios differing in terms of causal relatedness: unrelated, intermediately related, and highly related (similar to Keenan et al., 1984; see examples in Section 1.2), and found longer reading times and more temporal/inferior parietal/prefrontal hemodynamic activity in reading intermediately related sentences (compared to unrelated and highly related ones), which is the scenario eliciting elaborative causal inference. Such increases in reading times and hemodynamic responses are interpreted as reflecting the process of generating inferences when comprehenders try to build causal links between a target sentence and its contexts. Kuperberg et al. (2006) also suggested multiple brain areas involved in establishing causal inference (*cf.* Mason and Just, 2011), for example, inferior prefrontal regions that are engaged in the retrieval of semantic information, superior medial prefrontal regions involved in examining sequential relations between events, and right temporal and inferior prefrontal regions that are important for the detection of incoherence (p. 359).

### 5.3. Eye-movement measures

Eye movements can reveal how referents in mental models are retrieved and tracked (Altmann and Ekves, 2019). Eye-tracking provides a more continuous measure of comprehension than probe-recognition tasks and higher temporal sensitivity than brain activity measures like fMRI. Therefore, it enables the early processing of sentences to be closely tracked (Traxler et al., 1997).

#### 5.3.1. Online reading paradigms

Online reading paradigms are widely used to measure real-time comprehension of causality in texts. Reading times reveal readers’ processing loads while comprehending different linguistic elements that may depart from their expectations (Koornneef and Van Berkum, 2006; Featherstone and Sturt, 2010), and how their reading patterns are regulated by linguistic input such as causal connectives (Traxler et al., 1997; Canestrelli et al., 2013; Van Silfhout et al., 2014). Such measures provide a precise and comparatively natural measure of exact processing times for different regions of the sentence. They provide more direct and informative results on the time course of causal inference compared to retrieval paradigms.

Koornneef and Van Berkum (2006) used eye-tracking to investigate participants’ reading behaviors when comprehending implicit causality sentences (e.g., *David apologized/praised Linda because…*). The implicit causality verb in the first clause was manipulated in such a way that the referent of the following subordinate clause was either biased towards the first character David (by the verb *apologized*) or the second one Linda (by *praised*). During the two words that followed the pronoun of the subordinate clause under the inconsistent (vs. consistent) conditions, a reading delay emerged; and such a consistency effect was modulated by the verb type: The words after an inconsistent pronoun took longer to read with the NP2-biased verb (Koornneef and Van Berkum, 2006). The consistency effect influenced by the implicit causality cues emerged immediately after the pronoun (*cf.* Featherstone and Sturt, 2010) instead of in a delayed-integration fashion.

Given the benefits of immediacy, online reading methods cannot easily illuminate what people are attending to when they encounter delays in reading. For instance, researchers can gain more insights into the mechanisms of inferencing by examining what other content (beyond the currently heard/read words) is processed or receives attention during processing, and how such processes impact people’s mental representations. In addition, reading paradigms are less compatible with manipulations in the visual domain,^5^ making it difficult to explore the influence of visual information and the interplay of various sources of information. Therefore, methods that directly measure shifts in readers’ attention in visual contexts during language processing, such as the *visual world paradigm*, can compensate for the lack of information.

#### 5.3.2. Visual world paradigm

The visual world paradigm (VWP) uses eye-tracking to develop knowledge of causal inference by providing a window on mental representations and offers a way to explore how one or more of the elements comprising an event are represented and processed. On the one hand, measuring eye movement as a function of linguistic input spotlights how comprehenders focus on a particular target depending on the information they receive. For instance, studies of implicit causality have successfully shown that fixations on a visual character are influenced by verb cues (discussed in detail in Section 3.1; Pyykkönen and Järvikivi, 2010; Cozijn et al., 2011a) as well as discourse connectives (Section 3.2; Mak et al., 2013; Wei et al., 2019). On the other hand, visual contexts also affect language processing at different levels (Tanenhaus et al., 1995; Allopenna et al., 1998). Visual information about the relation between entities in an event, for example, facilitates the resolution of thematic role ambiguity (see Section 2 for details; Knoeferle et al., 2005).

Research applying this paradigm to investigate spoken language processing has revealed that the implicit causality effect occurs very rapidly and is expectation-based (Pyykkönen and Järvikivi, 2010; Cozijn et al., 2011a). Presenting two visual characters representing the NP1 and NP2 of the first clause in a visual world paradigm, Cozijn et al. (2011a) measured the proportion of looks to the two characters as listeners heard sentences containing implicit-causality verbs (e.g., NP1-biased: *The octopus bored the crocodile in the car because he*… and NP2-biased: *The camel felt sorry for the octopus after the exam because he*…). The findings showed that—straight after the connective *because* and the ambiguous pronoun *he*, and well before the disambiguation information (a follow-up clause clarifying the intended referent) was available—the verb type interacted with the character areas that listeners focused on. That is, the participants looked more at the NP1 than the NP2 referent if they heard the NP1-biasing verb in the first clause, whereas they focused more on the NP2 than NP1 referent in the NP2-biasing verb condition. Listeners started to exploit information in the implicit causality verb before they heard the disambiguating words in the subordinate clause (the point where information would be integrated). Using a similar VWP setting, Pyykkönen and Järvikivi (2010) observed an implicit causality effect immediately following the finite verbs. This proactive or *forward-looking* mechanism of using implicit causality information aligns with the discourse processing hypothesis that readers develop expectations about protagonist(s) as the discourse unfolds (Arnold, 2001).

Research on causal inference benefits from VWP eye-tracking in at least three ways. First, eye movements during processing can be precisely time-locked, which enables the online process of causal inference to be examined more closely. Second, measuring which areas of visual contexts receive the most attention (by observing participants’ looks at visual objects) generates evidence for the dynamics of mental representations that allow causes and consequences to be inferred. Importantly, the VWP can be used to explore how various sources of information, particularly non-linguistic visual information, affect causal inference. Some attempts have been made to directly investigate causal inference in an event-based visual context that prompted participants to observe depicted objects representing the cause/consequence of an event (Van Veen, 2011; Rohde and Horton, 2014; see details in Section 3.3). Nevertheless, much remains unexplored in terms of how various sources of cues affect causal inference in visual contexts with more situational elements, when such an influence takes place, and how links between events are established in more extensive causal chains.

## 6. Conclusion

This paper has reviewed the current state of knowledge regarding causal inference. Various sources of information contribute to causal inference, ranging from current linguistic input to non-linguistic visual context and real-world knowledge (e.g., event knowledge). Extending these sources to include non-linguistic and multifaceted representations of events will open up additional insights into causal inference based on event information, while also moving from isolated sentences to sequences of sentences and entire narratives corresponding to a mental world.

The article reviewed research on the process of causal inferencing, including inferences as to which protagonist is responsible for a cause or consequence (implicit causality), and inferences about the entire cause/consequence event. A method such as the visual world paradigm perhaps complementary with other measures may enable research on causal inference to move from focusing on inferences related to isolated objects to inferences about events and situations, which contain (but need not be limited to) referents, agents, patients, location, source, goal, etc. The recent development of *visual reality* techniques combined with eye-tracking (Eichert et al., 2018; Heyselaar et al., 2020) reflects this trend and offers new possibilities for investigating processing in an enriched visual context.

This review also examines the precise time course of causal inference which is crucial to comprehension from two perspectives, namely when coherence relations are built and when event-related information is accessed. Findings from these two lines of research suggest that methodology strongly influences the interpretation of results and accordingly the accounts that we postulate. Evidence from online measures, for example, supports the view that the processing of linguistic information and events is incremental and may be guided by expectations. In future studies, a combination of online and offline methods and rich contextual embedding could enable us to further refine accounts of (causal) inferences in language processing with specifications regarding the role of expectations as a function of specifics of a situation and of comprehenders.

## Author contributions

YW and PK contributed to the conception of the study. YW wrote the first draft of the manuscript. All authors contributed to the article and approved the submitted version.

## Funding

The study was supported by Ministry of Education in China – Project of Humanities and Social Science (No. 21YJC740062) granted to the first author.

## Conflict of interest

The authors declare that the research was conducted in the absence of any commercial or financial relationships that could be construed as a potential conflict of interest.

## Publisher’s note

All claims expressed in this article are solely those of the authors and do not necessarily represent those of their affiliated organizations, or those of the publisher, the editors and the reviewers. Any product that may be evaluated in this article, or claim that may be made by its manufacturer, is not guaranteed or endorsed by the publisher.

## Footnotes

^1^In this review, we use *expectation* as an umbrella term to cover both prediction in a general sense to refer to the state of language processing influenced by contextual information before new input is available (Kuperberg and Jaeger, 2016), and with the narrow sense of prediction used in probabilistic processing models (Feldman et al., 2009; Hale, 2011; Demberg et al., 2013).

^2^Rohde and Horton (2014), used the term *occasion* (which includes the category of *consequence*) instead. For simplicity’s sake, we do not distinguish between the two terms in the current review.

^3^The N400 component in the event-related brain potential is a negative-going wave with a peak around 400 ms after stimulus onset and tends to be largest over centro-parietal electrodes. It is usually larger for semantically incongruent / mismatching compared to congruent / matching input (e.g., Kutas and Hillyard, 1984; see also Kutas and Federmeier, 2011). The P600 is a positive-going wave around 600 ms after stimulus onset with larger mean amplitudes for structurally expected / licensed than unexpected / not licensed material (e.g., Osterhout and Holcomb, 1992; Hagoort et al., 1993).

^4^FMRI measures the blood-oxygen-level dependent (BOLD) signals when participants are performing various tasks (Amodio and Frith, 2006). The BOLD indexes the changes in blood flow and blood oxygenation triggered by neural activities (Hillman, 2014).

^5^Although in some multimedia learning studies designed to investigate the integration of textual and pictorial information, a picture is attached to reading texts (Alemdag and Cagiltay, 2018; Latini et al., 2020), such integrative processing *via* multimedia is beyond the current domain of discussion. Another exception is the sentence-picture verification paradigm which combines pictures and sentences and asks participants to verify their (in)congruence (Carpenter and Just, 1975; Tanenhaus et al., 1976; Knoeferle et al., 2011).
